# Cytochrome P450 2C19 Polymorphisms and Its Association With Major Adverse Cardiac Events in Post-coronary Intervention Patients on Clopidogrel in the Tertiary Care Center

**DOI:** 10.7759/cureus.34737

**Published:** 2023-02-07

**Authors:** Nikhil Teja Kambhampati, Hisham Ahamed, Velayudhan K.K, Sachin David, Sai chandra Hakeem, Gopalakrishna Pillai, Niveditha Kartha

**Affiliations:** 1 Internal Medicine, Amrita Institute of Medical Sciences and Research Centre, Kochi, IND; 2 Cardiology, Amrita Institute of Medical Sciences and Research Centre, Kochi, IND; 3 Genetics, Amrita Institute of Medical Sciences and Research Centre, Kochi, IND; 4 Biostatistics, Amrita Institute of Medical Sciences and Research Centre, Kochi, IND

**Keywords:** post-coronary intervention, coronary artery disease, clopidogrel, major adverse cardiovascular event, genetic polymorphisms

## Abstract

Background

Clopidogrel has become essential in managing coronary artery disease and other atherothrombotic diseases. It is an inactive prodrug that needs biotransformation in the liver by various cytochrome P (CYP) 450 isoenzymes for its active metabolite formation. However, 4-30% of patients on clopidogrel have shown no or decreased antiplatelet response. This condition is called ‘clopidogrel non-responsiveness’ or ‘clopidogrel resistance.’ This is attributed to genetic heterogeneity causing interindividual variation and increased risk of major adverse cardiac events (MACEs). This study aimed to assess MACEs and their association with CYP450 2C19 polymorphisms in post-coronary intervention patients on clopidogrel.

Methods

This prospective observational study was conducted on acute coronary syndrome patients, started on clopidogrel following coronary intervention. After considering inclusion and exclusion criteria, 72 patients were enrolled, and a genetic analysis was done. Based on genetic analysis, patients were divided into two groups, normal (CYP2C19*1) and abnormal phenotypes (CYP2C19*2 & *3). These patients were followed for two years, and the MACE during the first year and second year was compared between these two groups.

Results

Of 72 patients, 39 (54.1%) were normal, and 33 (45.8%) were abnormal genotypes. The mean age of patients is 67.71 ± 9.968. A total of 19 and 27 MACEs were seen during first- and second-year follow-ups. During the first-year follow-up, three (9.1%) patients with abnormal phenotypes developed ST-elevation myocardial infarction (STEMI), and none of the phenotypically normal patients developed STEMI (p-value = 0.183). Non-ST elevation myocardial infarction (NSTEMI) was seen in three (7.7%) normal and seven (21.2%) abnormal phenotype patients (p-value=0.19). Other events, such as thrombotic stroke, stent thrombosis, and cardiac death, were seen in two (6.1%) abnormal phenotypic patients (p-value=0.401). During the second-year follow-up, STEMI was seen in one (2.6%) normal and three (9.7%) abnormal phenotypic patients (p-value=0.183). NSTEMI was seen in four (10.3%) normal and nine (29%) abnormal phenotype patients (p=0.045). Comparison of total MACEs between normal and abnormal phenotypic groups at the end of the first year^ ^(p-value=0.011) and second year (p-value=<0.01) has statistical significance.

Conclusion

We can infer that the risk of developing a recurrent MACE in post-coronary intervention patients on clopidogrel is significantly high in the abnormal phenotypic group (CYP2C19*2 & *3) than in normal phenotypic patients.

## Introduction

As per the World Health Organization, cardiovascular diseases such as ischemic heart disease and cerebrovascular accidents, such as ischemic stroke, account for 17.7 million deaths and are the leading cause. One-fifth of these deaths were encountered in India [1]. Currently, timely percutaneous coronary intervention (PCI) has changed the outcomes of myocardial ischemia [2]. Unfortunately, even after PCI, there is a high risk of recurrent coronary artery disease (CAD), which is commonly encountered in patients with multiple comorbid conditions, like systemic hypertension (SHTN), type 2 diabetes mellitus (T2DM), renal diseases, and dyslipidemia (DLP) [3]. Platelet aggregation is the primary step in thrombosis and stent-related complications. Thus, antiplatelet therapy after PCI had become the cornerstone medical treatment. Clopidogrel (purinergic receptor (P2Y12) inhibitor) and aspirin are the most commonly used antiplatelets to decrease further cardiovascular events [4]. Clopidogrel is an inactive prodrug, and only 15% of its total consumption is absorbed. This absorbed drug undergoes biotransformation by various hepatic cytochrome P450 (CYP) isoenzymes to form the active metabolite [5]. These active metabolites will bind selectively and irreversibly to the P2Y12 receptor on the platelet surface, which will then inactivate the glycoprotein (GP) IIb/IIIa receptor and inhibit adenosine diphosphate-mediated platelet activation and aggregation. However, 4-30% of patients on clopidogrel have shown no or decreased antiplatelet response. This condition is called `clopidogrel non-responsiveness’ or ‘clopidogrel resistance.’ This will increase the risk of major adverse cardiac events (MACEs) [6,7]. 

This interindividual variability is mainly based on the pharmacodynamics of the drug. Various studies have shown gene polymorphism as one of the main reasons for clopidogrel resistance.

Among the various CYP450 isoenzymes, CYP2C19 plays an important role and contributes significantly to the metabolism of clopidogrel to its active form [8]. Different single nucleotide polymorphisms of the gene which encodes CYP2C19 isoenzyme have been reported, which include the CYP2C19 *2, *3, and *17 alleles. CYP2C19 *1 allele is a standard (wild-type allele) with complete enzymatic activity [9]. CYP2C19 *2 and *3 alleles are loss-of-function (LOF) variant alleles associated with decreased functional metabolic activity [10]. 

Few randomized controlled trials on the Asian population revealed a wide interindividual variability in CYP2C19 polymorphisms. Among Asians, about 55% are LOF-carrying alleles [11,12]. Therefore, individuals who carry LOF variant alleles will have low levels of active clopidogrel compared to non-carriers (CYP2C19 *1). Hence, they experience a high risk of thrombotic events [13]. 

However, the risk of thrombotic events is a multifactorial phenomenon commonly associated with comorbid disorders like T2DM, SHTN, and DLP and solely does not depend on P2Y12 inhibition [14]. Still, it is a matter of debate, whether these genetic variants that result in clopidogrel resistance have a synergistic impact on the risk of MACEs. In order to address this entity, pharmacogenomics is therefore useful. This study aimed to assess the MACE and its association with CYP450 2C19 polymorphisms in post-coronary intervention patients who are on clopidogrel.

## Materials and methods

This is a prospective observational study on acute coronary syndrome (ACS) patients who underwent PCI and started on clopidogrel as a maintenance dose at our tertiary care hospital (Amrita Institute of Medical Sciences and Research Centre, Kochi, Kerala, India) between September 2020 and 2022.

This study was performed as per the ethical standards laid down in the 1964 declaration of Helsinki and its later amendments or comparable ethical standards. Clearance was obtained from the institutional review board and the ethics committee before commencing the study (ECASM-AIMS-2020-404).

Based on the results of the proportion of myocardial infarction (MI) in the normal genotype (2.63%) and in the abnormal genotype (35.78%) (CYP450 2C19 polymorphisms) in post-coronary intervention patients on clopidogrel, observed in existing literature (Genetic polymorphisms in clopidogrel and its association with adverse cardiac events After Coronary Intervention In tertiary care center from south India. European Journal of Molecular & Clinical Medicine ISSN 2515-8260 Volume 07, Issue 03, 2020), and with 80 % power and 95% confidence, the minimum sample size comes to 21, each totaling 42 samples. 

Selection and description of participants

Based on inclusion and exclusion criteria, patients were enrolled in the study.

Inclusion Criteria

Patients of age more than 18 years were diagnosed with ACS (ST- Elevation Myocardial Infarction (STEMI) or Non- ST- Elevation Myocardial Infarction (NSTEMI)) during index hospitalization and started on clopidogrel post-PCI and given consent to follow-up for one year and two years were included in the study. 

Exclusion Criteria

Patients on other antithrombotic drugs, such as glycoprotein inhibitors and proton pump inhibitors like omeprazole, were excluded from the study. Patients with bleeding disorders or coagulopathies, cognition, or memory impairment, and having a life expectancy of less than 1 year were not included in the study. 

After considering above-mentioned inclusion and exclusion criteria, a total of 72 patients were enrolled in the study. Genetic analysis was done, and these 72 patients were divided into two groups, normal (CYP2C19*1) and abnormal (CYP2C19 *2& *3) phenotypic groups.

Data were collected from the hospital information system and simple questionnaires. The parameters included in the study are demographic data (age, gender, place), body mass index, personal habits (smoking and alcohol consumption), family history of CAD, other comorbid illnesses (chronic kidney disease (CKD), chronic liver disease (CLD), T2DM, SHTN, and DLP), the severity of the CAD, and MACEs. We also included data regarding the patients, who were managed with medical management (on antiplatelets, statins, and beta blockers) alone and patients who were managed by revascularization procedures and continued on medical management. 

MACEs recorded during the study were MI (STEMI and NSTEMI), thrombotic stroke, cardiac death, and stent thrombosis. These patients were followed up for two years from index presentation, and MACEs were recorded.

Genetic Analysis

**Figure 1:**
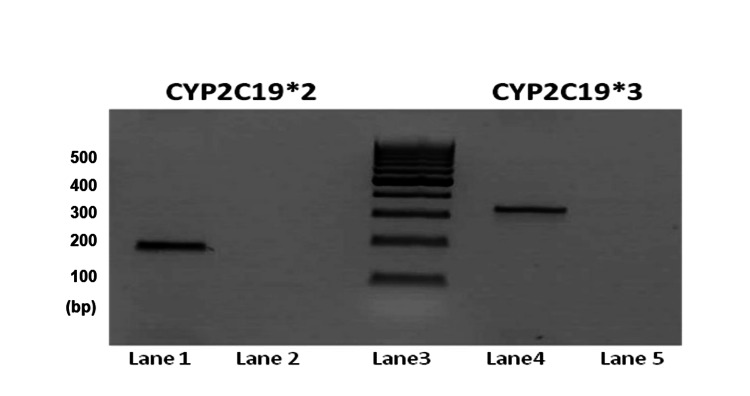
Polymerase Chain Reaction Products of CYP2C19*2 and CYP2C19*3 in 2% Agarose Gel Electrophoresis Lane 1: Polymerase chain reaction product of CYP2C19*2 of 168bp Lane2: Non-template control for CYP2C19*2 PCR Lane3: 100bp DNA ladder Lane4: Polymerase chain reaction product of CYP2C19*3 of 329bp Lane5: Non-template control for CYP2C19*3 PCR bp: Base pair, CYP: cytochrome P 450, DNA: deoxyribonucleic acid

Peripheral blood collected in tripotassium ethylenediaminetetraacetic acid (K3EDTA) tubes was used to extract deoxyribonucleic acid (DNA). The extraction was performed by the ethanol precipitation method. The pelleted-out DNA was dissolved in 1 X tris-EDTA (TE) buffer. Quantification of DNA was performed using BioPhotometer® D30 (Eppendorf, Hamburg, Germany). A ratio of ~2 for 260/280 and 1.8-2.2 for 260/230 was considered as good-quality DNA. The polymerase chain reactions (PCRs) for the polymorphism CYP2C19*2 and CYP2C19*3 were performed as separate PCRs. The PCR was performed in Veriti thermal cycler (Thermo Fisher Scientific, MA, USA) as per the PCR cycling conditions standardized in the laboratory. For PCR, 2% agarose gel electrophoresis (AGE) was performed to check the amplicons (Figure 1).

**Figure 2:**
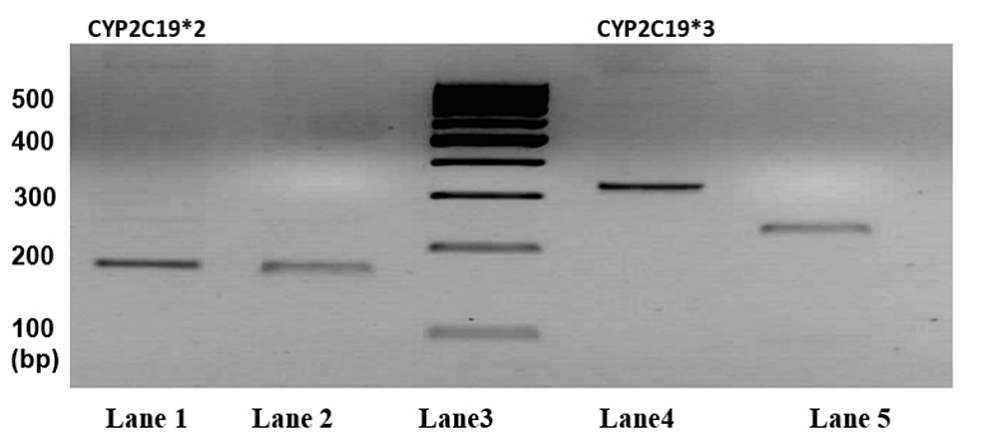
Representative Image of Restriction Length Fragment Polymorphism of CYP2C19*2 and CYP2C19*3 in 3% Agarose Gel Electrophoresis Lane 1: Polymerase chain reaction product of CYP2C19*2 of 168bp; Lane2: Digested product of CYP2C19*2 which shows 168bp identified as CYP2C19*2 variant Lane3: 100bp DNA ladder Lane4: Polymerase chain reaction product of CYP2C19*3 of 329bp Lane5: Digested product of CYP2C19*3 which shows 233 bp and 96bp identified as CYP2C19 and an absence of variant. bp: Base pair, CYP: cytochrome P 450, DNA: deoxyribonucleic acid

The gel was stained in ethidium bromide (EtBr) and was visualized in a Quantum gel documentation system (Vilber, Marne-la-Vallee Cedex, France). Restriction length fragment polymorphism was performed with enzymes SmaI and BamHI (Thermo Fisher Scientific, MA, USA) for CYP2C19*2 and CYP2C19*3, respectively, according to the manufacturer’s instructions, and the digested products were resolved at 3% AGE (Figure 2).

The presence of the CYP2C19*2 variant yielded 168 base pair (bp) sized products, and heterozygous yielded 168bp, 118bp, and 50bp, respectively. The wild type for CYP2C19 showed 118bp and 50bp when visualized in the gel. The CYP2C19*3 variant produced a product that was at 329bp, and a heterozygous variant produced 329bp, 233bp, and 96bp digested products. CYP2C19 wild type produced a product of 233bp and 96bp sizes.

Statistical Analysis

All data were compiled on an excel sheet, and statistical analysis was done using IBM SPSS Statistics for Windows, Version 20 (Released 2011; IBM Corp., Armonk, New York, United States). Numerical variables were represented using mean ± standard deviation (SD). Categorical variables were represented using numbers and percentages. To test the statistical significance of the difference in the proportion of STEMI, NSTEMI, thrombotic stroke, stent thrombosis, cardiac deaths, and the number of MACEs at the end of one year and second year between normal and abnormal phenotypes (CYP2C19*2 and CYP2C19*3), the Pearson Chi-Square test will be applied. A p-value of <0.05 was considered to be statistically significant. 

## Results

**Figure 3:**
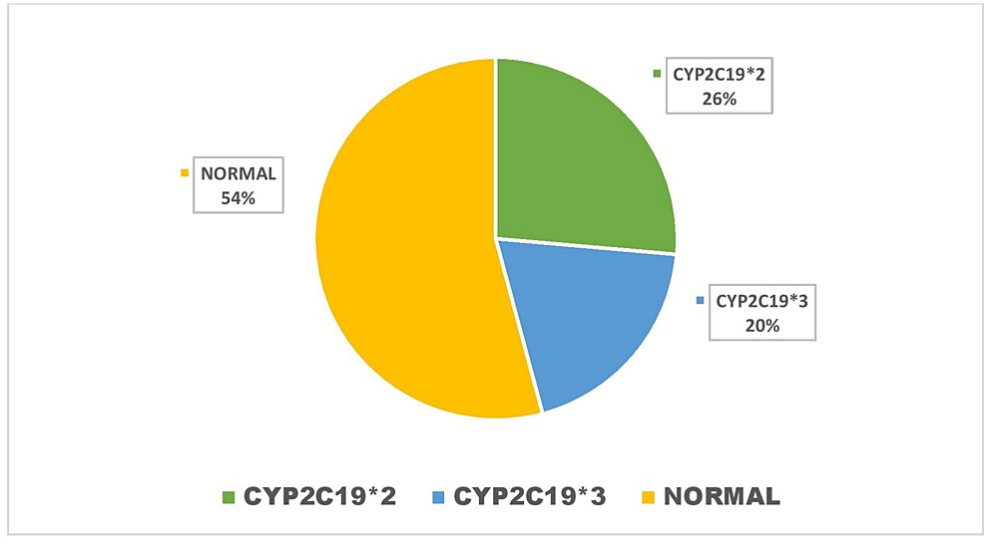
Genotype Distribution of the Study Population CYP: Cytochrome P 450

A total of 72 patients were enrolled in the study, and these patients were followed for two years. Genetic analysis was done, and based on the analysis, these patients were divided into two groups (normal and abnormal phenotypic groups). Of 72 patients, 39 had normal phenotypes, and 33 had abnormal phenotypes. Among abnormal phenotypes, 19 (26.3%) patients were found to have CYP2C19*2, and 14 (19%) had CYP2C19*3 phenotype (Figure 3).

Fourteen (56.9%) patients were male, and 31 (43.05 %) patients were females. The mean age of normal phenotypes is 69.97 ± 10.207 years, and in abnormal phenotypes, it is 65.03 ± 9.116 years. Risk factors like smoking was seen in 24 (33.3 %) patients, 23 (31.9%) patients had a habit of consuming alcohol, and 44 (61.11%) patients had a family history of CAD. T2DM, SHTN, and DLP were highly prevalent at 86%, 73.6%, and 97.2%. CKD and CLD were seen in 7(9.7%) and 20 (27.7%) patients. The ejection fraction (EF) < 40% was seen in 15 (20.8%) patients, 19 (26.3%) patients had single vessel disease, 28 (38.88%) patients had double vessel disease, and triple vessel disease was seen in 25 (34.7%) patients. PCI was undertaken in 41 (56.9%) patients, and 12 (16.6%) patients underwent coronary artery bypass graft. Medical management alone was given to 19 (26.38%) patients (Table 1).

**Table 1:** Baseline Demographics and Characteristics of the Study Population SD: Standard deviation, CAD: coronary artery disease, EF: ejection fraction, SVD: single vessel disease, TVD: triple vessel disease, PCI: percutaneous coronary intervention, CABG: coronary artery bypass graft

Demographic and clinical variables	Number of patients, n=72 (%)	Normal phenotypic group, n=39 (%)	Abnormal phenotypic group, n=33 (%)	p-value
Male, n (%)	41 (56.9)	22(56.4)	19(57.6)	0.921
Female, n (%)	31 (43.0)	17 (43.6)	14(42.4)	0.921
Age (years, mean ±SD)	67.71 ± 9.968	69.97 ± 10.207	65.03 ± 9.116	0.035
Obese n (%)	3(4.1)	1(2.6)	2(6.1)	0.478
Overweight, n (%)	13 (18.5)	8(20.5)	5(15.2)	0.478
Smoking, n (%)	24 (33.3)	13(33.3)	11(33.3)	1
Alcohol, n (%)	23 (31.9)	11 (28.2)	12 (36.4)	0.459
Family history of CAD, n (%)	44 (61.1)	20 (51.3)	24(72.7)	0.063
Type 2 diabetes mellitus, n (%)	62 (86.1)	34 (87.2)	28 (84.8)	0.776
Systemic hypertension, n (%)	53 (73.6)	27 (69.2)	26(78.8)	0.359
Chronic kidney disease, n (%)	20 (27.7)	13(33.3)	7(21.2)	0.253
Chronic liver disease, n (%)	7(9.7%)	5(12.8%)	2(6.1%)	0.572
Dyslipidemia, n (%)	70 (97.2)	37(94.9)	33(100)	0.549
EF < 40%, n (%)	15(20.8)	11(28.2)	4(12.1)	0.094
SVD, n (%)	19(26.3)	9(23.1)	10(30.3)	0.705
DVD, n (%)	28(38.8)	15(38.5)	13(39.4)	0.406
TVD, n (%)	25(34.7)	15(38.5)	10(30.3)	0.35
Medical management only, n (%)	19(26.3)	8(20.5)	11(33.4)	0.85
PCI, n (%)	41(56.9)	23(59.0)	18(54.5)	0.705
CABG, n (%)	12(16.6)	8(20.5)	4(12.1)	0.341

Outcomes

Patients were followed for two years, and outcomes were measured during first and second years. There were 19 MACEs during the first-year follow-up and 27 events during the second-year follow-up. During the first year, MI was seen in 13 patients, of which 3 (16%) were STEMI, and 10 (53%) were NSTEMI. Stent thrombosis, thrombotic stroke, and cardiac deaths were seen in two (10%) patients. During the second-year follow-up, MI was seen in 17 (24.2%) patients, out of which 4 (5.7%) were STEMI and 13(18.5%) were NSTEMI. Thrombotic stroke was seen in four (5.7%) patients. Stent thrombosis and cardiac deaths were seen in three (4.28%) patients (Table 2).

**Table 2:** Clinical Outcomes of the Study Population During the First and Second Years STEMI- ST- elevation myocardial infarction, NSTEMI- non-ST-elevation myocardial infarction

Major adverse cardiac events	During the first year	During the second year
STEMI	3(16%)	4(14.8)
NSTEMI	10(53%)	13(48.1)
Thrombotic stroke	2(10%)	4(14.8)
Stent thrombosis	2(10%)	3(11.1)
Cardiac deaths	2(10%)	3(11.1)

Comparison of MACEs Between Normal and Abnormal Phenotypes During the First-Year Follow-Up

The primary outcome of MI was more in the abnormal phenotype group than the normal phenotypic group. Among 72 patients, STEMI was seen in three (9.1%) abnormal phenotype patients, and none of the normal patients had STEMI during the first-year follow-up (p-value=0.450). But NSTEMI was seen in three (7.7%) normal and seven (21.2%) abnormal patients (p-value=0.045). Secondary outcomes like thrombotic stroke, stent thrombosis, and cardiac deaths were seen in two (10 %) of the abnormal phenotypic patients, and none of the normal patients had these events. In summary, during the first-year follow-up, six (18.2%) abnormal phenotypic patients and three (7.7%) normal patients had at least one event, and five (15.2%) abnormal phenotypic patients had two MACEs, but none of the normal phenotype patient has two MACEs. The comparison of total MACEs during the first year between normal and abnormal phenotypes was statistically significant (p-value=0.011) (Table 3).

**Table 3:** Association of Genetic Polymorphisms With Primary and Secondary Outcomes During the Second Year STEMI: ST- elevation myocardial infarction, NSTEMI: non-ST-elevation myocardial infarction, MACE: major adverse cardiac event

Major adverse cardiac events	Normal phenotype	Abnormal phenotype	p-value
STEMI	1(2.6)	3(9.7)	0.450
NSTEMI	4(10.3)	9(29)	0.045
Thrombotic stroke	0(0)	4(12.9)	0.073
Stent thrombosis	1(2.6)	2(6.5)	0.839
Cardiac deaths	0(0)	3(9.7)	0.164
Total major adverse cardiac events, n=27 (%)	6(22.2)	21(77.7)	<0.01

Comparison of MACEs Between Normal and Abnormal Phenotypes During the Second-year Follow-up

During the second year, STEMI was seen in one (2.6%) normal and three (9.7%) abnormal phenotypic patients. NSTEMI was seen in nine (29%) abnormal phenotypic patients and four (10.3%) normal patients, and comparison between these two groups in this particular entity has statistical significance (p-value=0.045). None of the normal phenotypic group had a thrombotic stroke, but 4 (12.9%) of the abnormal phenotypic group had a stroke. Stent thrombosis is seen in one (2%) normal and two (6.5%) abnormal phenotypic groups. Among abnormal phenotypic groups, there were three (9.7%) cardiac deaths, and no deaths were seen in the normal group. The comparison of total MACEs at the end of the second year between normal and abnormal phenotypes was statistically significant (p-value=<0.001) (Table 4).

**Table 4:** Association of Genetic Polymorphisms With Primary and Secondary Outcomes During the First Year STEMI- ST- Elevation myocardial infarction, NSTEMI- non-ST-elevation myocardial infarction

Major adverse cardiac events	Normal phenotype	Abnormal phenotype	p-value
STEMI	0(0)	3(9.1)	0.183
NSTEMI	3(7.7)	7(21.2)	0.19
Thrombotic stroke	0(0)	2(6.1)	0.401
Stent thrombosis	0(0)	2(6.1)	0.401
Cardiac deaths	0(0)	2(6.1)	0.401
Total major adverse cardiac events, n=19(%)	3(15.7)	16(84.21)	0.011

## Discussion

CAD is one of the leading causes of death worldwide. Management mainly includes medical and PCI. Despite many antiplatelet drugs in India, aspirin and clopidogrel are the main dual antiplatelets of choice because of affordability and availability factors. Even with the advancement in the management of CAD, many patients are still prone to recurrent cardiovascular events, and the incidence of recurrent MACEs is increasing. The reason for recurrent cardiovascular events was found to be multi-factorial. One of the hypotheses is variability in clopidogrel response. This variability was first discovered by Järemo et al., after which this entity received considerable attention [15]. Several studies were focused mainly on the genetic variability of clopidogrel metabolism, but very few studies were done on clopidogrel responsiveness and its association with the MACE. Our study aimed to determine the association of CYP2C19 polymorphisms and MACE during the first- and second-year follow-up.

This study was conducted among 72 patients admitted to a tertiary care center with MI and who underwent PCI. Among 72 patients, 39 (54.2%) were normal phenotypes, and 33 (45.8%) were abnormal phenotypes. Out of which, 41 were males, and 31 were females, with a mean age of around 65 years. These 72 patients were followed over two years, and the total numbers of MACEs during the first year and second year were compared between normal and abnormal patients. 

A total of 19 MACEs were found during the first-year follow-up, of which the majority were NSTEMI (10(53%)). Out of these 19 events, 16 were seen in abnormal phenotypic patients. Among these 16 events, six patients had at least one event, and five patients were found to have two events. This association of MACEs between normal and abnormal phenotypes showed statistical significance with a p-value of 0.011. Similarly, a retrospective study by Yu et al. showed higher LOF allele frequency in the MACE group than in the no-MACE group [16].

The incidence of NSTEMI and STEMI during the first year was compared between normal and abnormal phenotypes; 3 (9.1%) STEMIs, and 7 (21.2%) NSTEMIs were seen in the abnormal phenotypic group. In the normal phenotypic group, none of the patients had STEMI, but three (7.7%) patients had NSTEMI. Even though there is no statistical significance, the incidence of MI (STEMI and NSTEMI) is higher in the abnormal group. The study by Oh et al. also reported that stent thrombosis and MI were detected more commonly in CYP2C19*2 carriers than non-carriers [17].

Very few trials followed patients for two years and reported MACEs. In our study, we followed patients for two years and assessed MACEs during these two years. A total of 28 MACEs were encountered during the second year, of which seven events were seen in normal phenotypes, and 21 events were in abnormal phenotypes. This has a statistical significance with a p-value of <0.001. Among the 28 MACEs, 13 (46.45%) were NSTEMIs, of which 9 (69%) of them were seen in abnormal phenotypes (p-value = 0.045).

Studies conducted by Yu et al. [16] and Oh et al. [17] showed a significant incidence of MI at the end of one year in an abnormal phenotypic group, but few studies like those by Moa et al. [18] and Tang et al. [19] had reported no statistical heterogeneity in incidence of MI at the end of the first year, which was supporting the current study. But our study showed a significance in the incidence of MI during the second year of follow-up.

A total of four (5.5%) cardiac deaths were encountered during the study, of which two were seen during the first year, and the other two were during the second year. All these deaths were seen in the abnormal phenotypic group. Of 33 abnormal phenotypic patients, 19 (57%) had CYP2C19*2, and 14 (43%) had CYP2C19*3. At the end of one year, among these 19 CYP2C19*2 patients, six patients had one MACE, and one patient had two MACEs, whereas in the CYP2C19*3 group, four (28.6%) patients had two MACEs. The association of MACEs at the end of one year between CYP2C19*2 and CYP2C19*3 was statistically significant.

Many studies, like the PLATelet inhibition and patients Outcomes (PLATO) trial [20] and TRial to assess Improvement in therapeutic outcomes by optimizing platelet inhibitioN with prasugrel-Thrombolysis In Myocardial Infarction (TRITON-TIMI) 38 trial [21], had focused on comparing the incidence of cardiovascular events between clopidogrel and other antiplatelet drugs. The PLATO trial compared cardiovascular events between ticagrelor and clopidogrel and found that 9.8% of the ticagrelor group and 11.7% of the clopidogrel group had cardiovascular events [20]. However, the ticagrelor group showed increased survival rates, but a higher rate of major bleeding was noted.

Similarly, the TRITON-TIMI 38 trial compared prasugrel and clopidogrel, which reported that prasugrel is more effective than clopidogrel in preventing thrombotic events in patients with STEMI undergoing PCI [21]. Despite the fact that ticagrelor and prasugrel perform better than clopidogrel in terms of outcomes, clopidogrel is still the primary antiplatelet of choice because of its affordability, accessibility, and less risk of bleeding.

Hence, genetic-guided antiplatelet therapy would be more beneficial in patients receiving clopidogrel to prevent further cardiovascular events. This would be ideal in patients with LOF alleles on genetic analysis and expecting decreased antiplatelet response, where other antiplatelets should be considered.

Study limitations

Study Design

One of the main limitations of the study is the small sample size, which can underpower or overpower the findings. The ethnicity of the patients was not considered, whereas many other studies showed that ethnicity has a role in genetic heterogeneity.

Non-genetic Risk Factors

There are many non-genetic risk factors like drug-to-drug interaction, non-compliance, diet, and other demographic factors. All of these factors, which had an interaction with the metabolism of clopidogrel, were not considered. Consideration of the above factors in the study can alter the outcomes.

Genetic Variations of Other Genes

This study is limited to *2, *3 alleles of CYP2C19. Even though other alleles are not significant, the complete importance of other alleles still needs to be clarified. Assessment of other alleles might improve the significance of the study. This study is also limited only to CYP2C19 isoenzymes. Other genes like ABCB1, CYP1A2, CYP3A4, etc., involved in absorption and metabolism were not studied in the current study.

Platelet Function Assessment

A few literature studies showed a significant correlation between platelet function assessment and CYP2C19 pleomorphisms. Because of the limitation of funds, we could not assess platelet reactivity (platelet function tests) and relate to the heterogeneity of CYP2C19 isoenzymes.

## Conclusions

In conclusion, the present study showed the relationship between CYP2C19 polymorphisms and MACEs in CAD patients who were taking clopidogrel as a maintenance medication. From our findings, we can infer that the risk of developing recurrent MI (STEMI and NSTEMI) and other MACEs during one and two years of post-PCI is significantly higher in the abnormal phenotypic group (CYP2C19*2 & *3) than in normal patients. The PLATO trial and TRITON-TIMI 38 trial also demonstrated that the patients on clopidogrel had increased cardiovascular events compared to other P2Y12 inhibitors. But other P2Y12 inhibitors like prasugrel and ticagrelor have an increased risk of bleeding, and many other factors like affordability and availability of the drug can limit its compliance. So, we recommend phenotypic analysis prior to initiating clopidogrel therapy to reduce the risk of MACEs. 
